# Combining a Bilateral Bipedicle Flap and Artificial Dermis for Extensive Coverage in Meningomyelocele Surgery

**DOI:** 10.7759/cureus.50141

**Published:** 2023-12-07

**Authors:** Junya Oshima, Kaoru Sasaki, Yukiko Aihara, Mitsuru Sekido

**Affiliations:** 1 Department of Plastic, Reconstructive, and Hand Surgery, University of Tsukuba, Tsukuba, JPN

**Keywords:** soft tissue reconstruction, artificial dermis, flap, kyphosis, myelomeningocele

## Abstract

Myelomeningoceles with soft tissue defects are often difficult to close primarily and require reconstructive surgery. Furthermore, cases with large skin defects or cases with kyphosis of the spine require a large area to be covered, making reconstruction even more difficult. We performed closure of soft tissue defects using a bilateral bipedicle flap and application of artificial dermis to the flap donor area in three cases in which surgery was difficult. The bilateral bipedicle flap was able to easily and reliably close the soft tissue defect even in highly difficult emergency myelomeningocele surgery. We believe that applying artificial dermis to the flap donor area is a useful method that avoids autologous skin grafting and facilitates wound management. There have been no cases of major donor wound contracture. The healing period of the flap donor area may be predicted to some extent by the width immediately after surgery.

## Introduction

Meningomyelocele is the most common form of neural tube defect [1]. Myelomeningoceles with soft tissue defects are often difficult to close temporarily and require reconstructive surgery [2]. Furthermore, cases with large skin defects or cases with kyphosis of the spine require coverage of a large area, making reconstruction even more difficult. Numerous flap techniques have been described to repair meningomyelocele with soft tissue defects, but the optimal surgical technique is still being debated [3,4]. Here, we report on the reconstruction of soft tissue in myelomeningocele performed at our hospital using a bilateral bipedicle flap and artificial dermal covering for the donor area of the flap.

## Case presentation

Case 1

**Figure 1:**
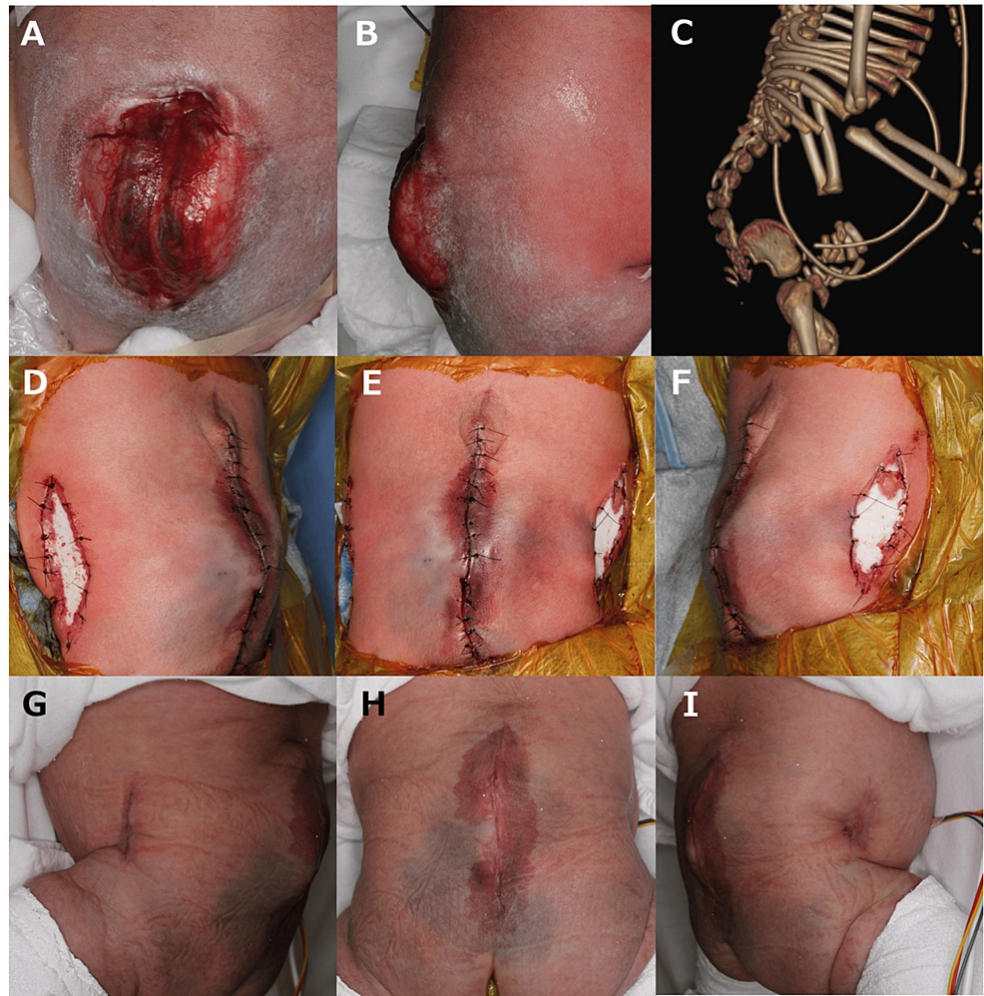
Case 1. (A) A skin defect measuring 4 cm x 6 cm was found on the lower back. (B) A 1.5-cm posterior prominence extending from the surface of the back. CT revealed severe kyphosis. After the skin defect was closed with a bilateral bipedicle flap, an artificial dermis was applied to the donor area: (D) left side, (E) front, and (F) right side. At 1.5 months after surgery, the progress was good: (G) left side, (H) front, and (I) right side.

A fetal diagnosis of myelomeningocele was made. The baby was born by cesarean section at 38 weeks' gestation, with a birth weight of 2,034 g. A skin defect measuring 4 cm x 6 cm was found on the lower back. CT revealed severe kyphosis and a 1.5 cm posterior prominence extending from the surface of the back. Surgery was performed one day after birth. After the neurosurgeon closed the dura, we made bilateral ventral midline incisions and harvested the bipedicle flap on both sides. The size of the skin flap was 4.5 cm x 4.5 cm. After confirming the reduction of tension in the midline, the two layers of the dermis and epidermis were sutured. Terdermis® was applied to the flap donor area. The minor axis width of the flap donor area was 1.2 cm on the left and 1.5 cm on the right. The film of the artificial dermis was removed on the 14th postoperative day, and thereafter, disinfection, external application of ointment, and gauze protection were performed every one to two days. The left flap donor area healed 31 days after surgery, and the right flap donor area healed 38 days after surgery. At 1.5 months after surgery, the postoperative course was good (Figure 1).

Case 2

**Figure 2:**
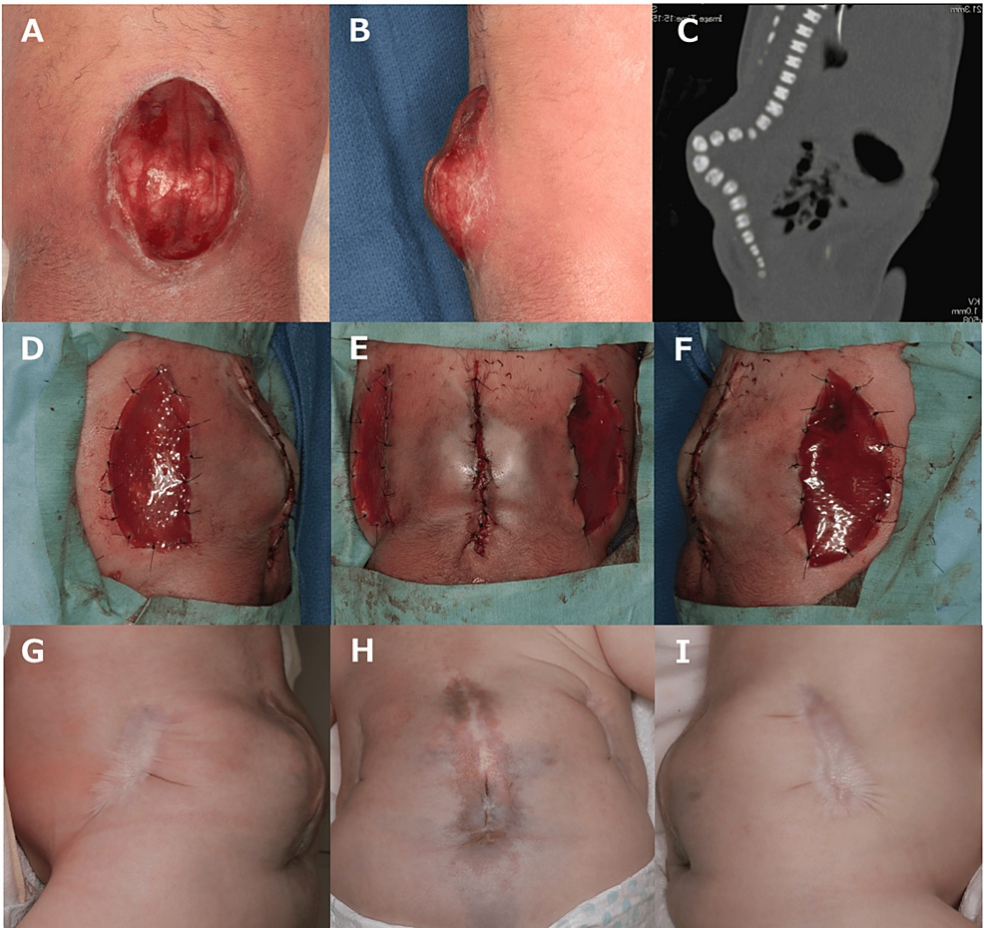
Case 2. (A) A skin defect measuring 4 cm x 6 cm was found on the lower back. (B) A 2-cm posterior prominence extending from the surface of the back. CT revealed severe kyphosis. After the skin defect with a bilateral bipedicle flap, an artificial dermis was applied to the donor area: (D) left side, (E) front, and (F) right side. At seven months after surgery, the progress was good: (G) left side, (H) front, and (I) right side.

The baby was born by cesarean section at 35 weeks’ gestation, with a birth weight of 2,374 g. A skin defect measuring 4 cm x 6 cm was found on the lower back. CT revealed severe kyphosis and a 2 cm posterior prominence extending from the surface of the back. Surgery was performed jointly with a neurosurgeon on the day of birth. We closed the midline defect with a 4.5 cm x 7.5 cm bilateral bipedicle flap. Integra® was applied to the flap donor area. The minor axis width of the flap donor area was 3 cm on both sides. The film of the artificial dermis was removed on the 14th postoperative day, and the same treatment as that for Case 1 was performed. Seven months after surgery, the postoperative course was good (Figure 2).

**Figure 3:**
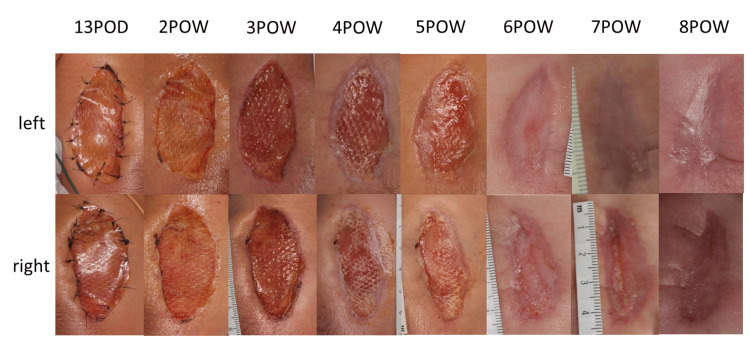
Postoperative course of artificial dermal covering in Case 2. The wound to which the artificial dermis was applied underwent smooth epithelialization and contraction from the surrounding area, and wound healing was achieved eight weeks after surgery. POD, postoperative day; POW, postoperative week

In this case, photographs of the artificial dermis were recorded, so images of the progress are presented. The wound to which the artificial dermis was applied underwent smooth epithelialization and contraction from the surrounding area, and wound healing was achieved 8 weeks after surgery (55 days after surgery) (Figure 3).

Case 3

**Figure 4:**
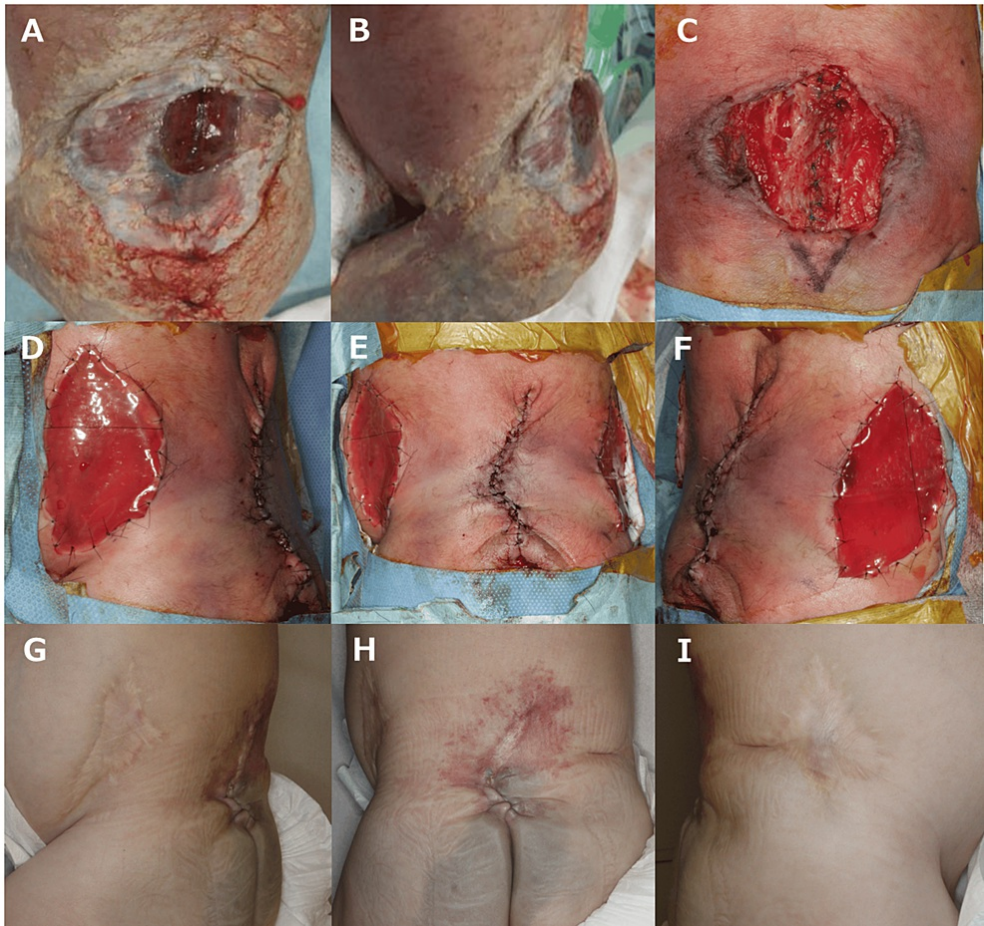
Case 3. Immediately after birth, there was a thin capsule on the surface, and it was vulnerable to closure of the meningocele: (A) frontal side and (B) lateral side. (C) When the thin capsule was removed, a 5 cm x 5 cm skin defect was observed. After the skin defect was closed with a bilateral bipedicle flap, an artificial dermis was applied to the donor area: (D) left side, (E) front, and (F) right side. At 15 months after surgery, the progress was good (G) left side, (H) front, and (I) right side.

The baby was born by cesarean section at 37 weeks’ gestation, with a birth weight of 3,295 g. A skin defect measuring 5 cm x 5 cm was found on the lower back. Surgery was performed jointly with a neurosurgeon on the day of birth. We closed the midline defect with a bilateral bipedicle flap. The flap size was 4.5 cm x 7.5 cm on the right and 4.5 cm x 8.5 cm on the left. Integra® was applied to the flap donor area. The minor axis width of the flap donor area was 4 cm on both sides. The film of the artificial dermis was removed on the 14th postoperative day, and the same treatment as that for Cases 1 and 2 was performed. Healing of the flap donor area was achieved 84 days after surgery. Fifteen months after surgery, the postoperative course was good (Figure 4).

A summary of the cases is shown in Table 1.

**Table 1:** A summary of the cases. POD, postoperative day

	Case 1		Case 2		Case 3	
Gestational age	38 weeks		35 weeks		37 weeks	
Birth weight	2,034 g		2,374 g		3,295 g	
Minor axis width of skin defect	4 cm		4 cm		5 cm	
Major axis width of skin defect	6 cm		6 cm		5cm	
Prominence of meningocele	1.5 cm		2 cm		0.5 cm	
Spinal kyphosis	55 degrees		68 degrees		None	
	Left	Right	Left	Right	Left	Right
Flap size (width x length)	4.5 cm × 4.5 cm	4.5 cm × 4.5 cm	4.5 cm × 7.5 cm	4.5 cm × 7.5 cm	4.5 cm × 7.5 cm	4.5 cm × 8.5 cm
Donor width immediately after surgery	1.2 cm	1.5 cm	3.5 cm	3.5 cm	4 cm	4 cm
Donor healing period	31 POD	38 POD	55 POD	55 POD	84 POD	84 POD
Artificial dermis used	Terdermis®		Integra®		Integra®	
Postoperative observation period	1.5 months		7 months		15 months	

## Discussion

Surgical treatment of myelomeningoceles is early skin closure, but in approximately 25% of cases, single-stage closure is not possible and plastic surgical intervention, including reconstructive surgery, is required [5]. Specific surgical techniques in terms of surgical time, bleeding, reliability, invasion, and durability have been reported, but the optimal surgical technique is still being debated. In particular, it has been reported that cases with spinal kyphosis or cases with skin defects of 5 cm or more in width have a large area to cover and are difficult to reconstruct [6,7].

The bilateral bipedicle flap for myelomeningoceles is a surgical technique that has been reported since the 1970s. Although the bipedicle flap can be harvested as a skin flap, we have harvested it as a myocutaneous flap that includes the latissimus dorsi and gluteus maximus muscles to ensure more stable blood flow [8,9]. It is a simple and reliable surgical method with stable blood flow and can be used even in cases with a high degree of difficulty in reconstruction [10,11]. In our cases, the patients of Cases 1 and 2 had severe spinal kyphosis, and the patient of Case 3 had a skin defect width of 5 cm, all of which were difficult to cover, but we were able to securely close the wound without excessive tension. In addition, because detailed preoperative studies such as skin blood flow and flap design were not required, emergency surgery could be performed on the first day of life. However, the disadvantage of the bilateral bipedicle flap is that it requires autologous skin grafting at the flap donor site [10]. Therefore, we applied artificial dermis to the flap donor for secondary healing.

Reports of the use of artificial dermis for myelomeningoceles are limited. The use of Terdermis® for temporary coverage before flap closure has been reported [12], as has the use of an acellular dermal matrix for temporary coverage until reoperation after flap closure fails [13]. However, the use of artificial dermis for flap donor wounds has not been reported. The use of artificial dermis for the donor wound allowed the avoidance of autologous skin grafting. Covering the donor wound reduces exudate from the wound, thereby reducing fluid loss in extremely underweight infants. In addition, daily care for wounds with artificial dermis is easier than for open wounds, and pain relief during care can be expected [14].

The method of secondary wound healing using only artificial dermal coverage is common for adult fingertips and small defects on the face [15,16]. However, the method has not been reported for relatively large defects of about 4 cm in width or newborns, presumably because the degree of contracture deformity and the treatment period are unknown. In our cases, the wounds to which the artificial dermis was applied took a certain period to heal, but all did so without infection or contracture. Regarding the healing period, a correlation was found between the width of the donor wound immediately after surgery (1.2-4 cm) and the healing period (31­­­-84 days). Although the differences depending on the type of artificial dermis are unknown owing to the small number of cases, it is possible that the healing period can be predicted to some extent by the width of the donor wound immediately after surgery. The disadvantage of using artificial dermis is that it requires a certain amount of cost and that preparations must be made in advance at the hospital. The limitations of this study are that it is a retrospective evaluation of a single surgical procedure and that it examines the short-term course.

## Conclusions

The bilateral bipedicle flap was able to easily and reliably close the midline defect even in highly difficult emergency myelomeningocele surgery. We believe that applying artificial dermis to the flap donor site in a bilateral bipedicle flap is a useful method that avoids autologous skin grafting and facilitates wound management. There have been no cases of major donor wound contracture. The healing period of the flap donor area may be predicted to some extent by the width immediately after surgery.

## References

[1] Putri NM, Tunjung N, Sadikin PM (2021). Closure of meningomyelocele defects using various types of keystone-design perforator island flaps. Arch Plast Surg.

[2] Duffy FJ Jr, Weprin BE, Swift DM (2004). A new approach to closure of large lumbosacral myelomeningoceles: the superior gluteal artery perforator flap. Plast Reconstr Surg.

[3] Kattan AE, Alsufayan FA, Alammar AK, Alhazmi B, Ahmed A, Gelidan AG, Almishal OM (2020). Extended transverse-oblique back flap for myelomeningocele defect closure: a case series of 10 patients. Plast Reconstr Surg Glob Open.

[4] Mortada H, Alhablany T, A Bhat T, Al Tamimi A (2021). Closure of a large myelomeningocele defect using the V-Y rotation advancement flap (butterfly flap): a case report and literature review. Case Reports Plast Surg Hand Surg.

[5] PA TJ (1959). The use of rotation flaps following excision of lumbar myelo-meningoceles: an aid to the closure of large defects. Br J Surg.

[6] Duddy JC, Caird J, Connolly P (2013). Repair of a large thoracolumbar myelomeningocele with associated lumbar kyphosis. Acta Neurochir (Wien).

[7] Kocak OF, Demir CY (2016). An ideal flap alternative for closure of myelomeningocele defects: dorsal intercostal artery perforator flap. J Craniofac Surg.

[8] McDevitt NB, Gillespie RP, Woosley RE, Whitt JJ, Bevin AG (1982). Closure of thoracic and lumbar dysgraphic defects using bilateral latissimus dorsi myocutaneous flap transfer with extended gluteal fasciocutaneous flaps. Childs Brain.

[9] Moore TS, Dreyer TM, Bevin AG (1984). Closure of large spina bifida cystica defects with bilateral bipedicled musculocutaneous flaps. Plast Reconstr Surg.

[10] Ramirez OM, Ramasastry SS, Granick MS, Pang D, Futrell JW (1987). A new surgical approach to closure of large lumbosacral meningomyelocele defects. Plast Reconstr Surg.

[11] El-khatib HA (2004). Large thoracolumbar meningomyelocele defects: incidence and clinical experiences with different modalities of latissimus dorsi musculocutaneus flap. Br J Plast Surg.

[12] Nakazawa H, Kikuchi Y, Honda T, Isago T, Nozaki M (2005). Successful management of a small infant born with a large meningomyelocele using a temporary artificial dermis. Scand J Plast Reconstr Surg Hand Surg.

[13] Susarla SM, Hauptman J, Ettinger R, Sittler B, Ellenbogen RG (2019). Acellular dermal matrix as a definitive reconstructive option for management of a large myelomeningocele defect in the setting of severe lumbar kyphosis. World Neurosurg.

[14] Bessho K, Murakami K, Iizuka T (1998). The use of a new bilayer artificial dermis for vestibular extension. Br J Oral Maxillofac Surg.

[15] Lou X, Zhu H, Xue H, Weng Y, Chen J (2018). One-stage wound healing of fingertip injuries induced by treatment of artificial dermis. Handchir Mikrochir Plast Chir.

[16] Han SK, Kim SY, Choi RJ, Jeong SH, Kim WK (2014). Comparison of tissue-engineered and artificial dermis grafts after removal of basal cell carcinoma on face--a pilot study. Dermatol Surg.

